# Social care occupational therapy and learning disabilities: professional perspectives on communication, collaboration and making a difference

**DOI:** 10.1080/17482631.2026.2721738

**Published:** 2026-09-08

**Authors:** T. Swinson, B. Littlechild, G. Howcroft, K. McNamara, S. Prowse, B. White, K. Almack

**Affiliations:** a East Herts Adult Disability Team, Hertfordshire County Council, Stevenage, UK; b CRIPACC, University of Hertfordshire, Hatfield, UK; c Health Liaison Team, Hertfordshire County Council, Stevenage, UK; d Adult Care Services, Hertfordshire County Council, Stevenage, UK; e Independent Expert by Experience, Norfolk, UK

**Keywords:** Occupational therapy, social care, learning disabilities, carers, intellectual disabilities, informal care, unpaid care

## Abstract

**Purpose:**

Adults with learning disabilities experience a poorer quality of life than the general population. Social care can critically support such adults and their informal carers, but limited literature exists about occupational therapy provision. This study explored the extent to which social care occupational therapists support communication and collaboration, and make a difference.

**Methods:**

Eight social care occupational therapists were recruited through professional networks. Online interviews were undertaken using topic guides co-designed with professionals and experts by experience. Reflexive thematic analysis was employed, informed by critical realism.

**Findings:**

Therapists pursued inclusive practices with adults with learning disabilities and valued informal carers. The communication challenges increased the reliance on carer input and raised doubts about interventions reflecting the wishes of adults with learning disabilities. Carer disagreements were navigated through diplomacy and positive risk-taking, but concerns about moving and handling safety led to faltering collaboration. Therapists felt that they enhanced community engagement and reduced carer burden, but workload limited their scope of practice.

**Conclusions:**

Social care occupational therapists strive to work inclusively with adults with learning disabilities and their informal carers, with beneficial outcomes, requiring various profession-based and interpersonal skillsets. However, communication difficulties create uncertainty. Practice-improvement opportunities include additional training and enhanced multidisciplinary working.

## Introduction

This paper reports on a small-scale qualitative study within the East of England, which highlights the ways in which social care occupational therapists work with adults with learning disabilities and their informal carers, and perspectives on the impact they may have as part of the occupational therapy process.

There are currently 1.2 million adults with learning disabilities living in the UK (Mencap, 2026). A learning disability diagnosis is characterized by a lower intellectual ability (IQ lower than 70), variable but significant impairment of social or adaptive functioning, and the onset of such factors in childhood (American Psychiatric Association, 2013). An increased severity of learning disability increases the likelihood of impairments in communication, skill learning, and undertaking activities of daily living (Care, 2015).

In the UK, adults with learning disabilities continue to experience poorer health and quality of life compared to the general population (National Institute for Health & Care Excellence [NICE, 2021). Occupational therapists possess expertise in promoting participation in activities of daily living, making them well-placed to improve health and well-being outcomes for this population (Royal College of Occupational Therapists [RCOT], 2019). However, there is still limited research which explores this provision (Haines et al., 2018). This is particularly the case within social care research, despite social care services forming a significant part of the infrastructure for supporting people with learning disabilities. From 2020 to 2021, 46% of people aged 18–64 receiving long-term support had learning disabilities as their primary support reason (NHS Digital, 2021). In England and Wales, the legislative framework set out in the Care Act 2014 and c. 23 (2014) mandates that the primary role of social care is to help people “achieve the outcomes that matter to them in their life” (Department of Health, 2022 sec.1.1). The pursuit of these outcomes also importantly extends to informal carers, who make up a substantial and critical unpaid workforce for adults with learning disabilities (Carers UK, 2019; Department of Health & Social Care, 2021; Idriss et al., 2020; NHS Digital, 2019). Most adults with learning disabilities do not live in residential care (Nuffield Trust, 2022), with two-thirds of adults still living in the family home (Care, 2018).

To the authors’ knowledge, no published research specifically examines social care occupational therapy provision for these groups. Statutory guidance recognizes social care occupational therapists as an essential profession (Local Government Association, 2025), which is tasked to enhance the well-being of individuals (and their carers) by helping them overcome difficulties in their everyday activities (RCOT, 2026). In practice, this could include the provision of information and advice, developing skills and confidence, moving and handling assessments, equipment provision, minor and major adaptations to people’s homes, and writing housing needs reports that highlight the accommodation needs of an individual.

Demonstrating the impact of occupational therapy is a stated research priority for, and working with families, including informal carers, is recognized as a core component of occupational therapy practice (Learning Disabilities Professional Senate, 2014; Lillywhite & Haines, 2010). RCOT further highlights the need to strengthen the effectiveness of work with families and carers within its research agenda (RCOT, 2020). Engaging carers in research not only provides perspectives that complement or substitute for those clients who may be unable to articulate their own views (Pickard & Knight, 2005) but also generates evidence on how best to support carers’ needs—an area that remains under-researched within learning disabilities (Henwood et al., 2019).

The study reported in this paper forms part of the Social Care Research in Practice (SCRiPT) study (Lynch et al., under review), where four Research in Practice Teams (RiPTs) were established; designed to build the research capacity of social care professionals, with the important inclusion of experts by experience (Warmoth et al., 2025; Woolham et al., 2025). This approach enables early and meaningful involvement from key stakeholders and supports research conducted *with* rather than *about* or *to* those affected (Health Research Authority, 2022). Such participation, which is still uncommon for adults with learning disabilities and informal carers, can offer valuable insights for the research team (Beighton et al., 2019).

The RiPT reported in this paper included adults with learning disabilities, informal carers, and professionals with expertise in social care, occupational therapy and social work. The team identified a need to explore the impact of social care occupational therapy on the lives of adults with learning disabilities and their informal carers, and the communication and collaboration that is part of this process. This focus responds directly to documented concerns of poor communication between health and social care professionals and adults with learning disabilities and their informal carers (Mencap, 2012), with research suggesting that staff can lack knowledge about communication needs and how these can be addressed (Bradshaw, 2020). It is particularly noteworthy given that almost 60% of adults with learning disabilities have communication problems (Smith et al., 2020), and such difficulties, including the expression and understanding of speech, are linked to a lower quality of life (García et al., 2020). The importance of facilitating communication is enshrined in law in the UK Equality Act (2010) under reasonable adjustments, and forms a core tenet of professional occupational therapy standards, which emphasize personalized communication, shared decision making and partnership working (Royal College of Occupational Therapists, 2021).

To address the lack of empirical literature in the highlighted areas, this study addresses the following questions:

(1) *To what extent do social care occupational therapists support communication and collaboration with adults with learning disabilities and their informal carers as part of the occupational therapy process?*
(2) *To what extent do social care occupational therapists make a difference to the lives of adults with learning disabilities and informal carers?*

## Method

### Design

This study was a pragmatic qualitative exploratory study, allowing a focus on research questions informed by practice within the context of limited existing research. It was originally envisaged to collect perspectives from adults with learning disabilities, informal carers and social care occupational therapists. However, owing to difficulties with recruiting all three groups within the timeframe for data collection, the decision was collectively made to focus on professional perspectives—where there had already been some limited interest—and interviews, via MS Teams, were chosen as a pragmatic solution, unless reasonable adjustments would require otherwise (e.g., doing an interview face-to-face). Semi-structured interviews were identified as the most appropriate format, allowing the collection of perspectives around some prepared questions, with the flexibility to broach into other questions as a result of the conversation (DiCicco-Bloom & Crabtree, 2006). The interview topic guides were co-designed with professionals and expert by experience colleagues in the research in practice team, focusing on the areas of communication, collaboration and making a difference to people’s lives.

### Recruitment

Social care occupational therapists were purposively sampled, with efforts to recruit staff who were practising social care occupational therapists with relevant, recent experience working with adults with learning disabilities and their informal carers. Attempts to recruit occupational therapists for interviews were undertaken in the first instance via electronic correspondence to occupational therapist leadership within multiple local authorities. Two principal occupational therapists engaged with the communication. One principal occupational therapist attempted to involve their colleagues; however, they were informed that occupational therapist time could not be utilized for the research because of low staff resource levels. Another Principal Occupational Therapist supported recruitment through a number of activities within their organization; using email correspondence with appropriate occupational therapy colleagues, and promoted the research at relevant senior-level meetings and occupational therapy continuing professional development meetings; they also allowed the lead researcher to write a piece in an email briefing and present at the county-wide meeting for occupational therapists. Eight occupational therapists contacted TS to express their interest in participating. All had experience in working as social care occupational therapists with adults with learning disabilities and their informal carers, but they varied in their geographical location (and teams) within the local authority, seniority, sex, and ethnicity; however, given the small sample, these are not described in any detail to protect confidentiality. Most participants were white, female, and their experience ranged from those at the start of their occupational therapy career to those with many years of experience. For context, their remit often involved working with adults with severe learning disabilities who also had physical disabilities.

### Procedure

Individual, semi-structured interviews took place over Microsoft Teams, and a voice recorder was used by the interviewer (TS); this allowed two recordings (MS Teams and the voice recorder) to take place in case one failed. In all but one case (due to voice recorder failure), voice recorder files were used for transcription, as this avoided the need for video files to be transferred unnecessarily.

### Data analysis

Audio or video files were transcribed by a professional transcriber who had signed a non-disclosure agreement to promote confidentiality. These files were then imported into NVivo 12 Plus to assist in data management. The interview data were then analyzed adopting the process of reflexive thematic analysis (Braun & Clarke, 2022), undertaken by the lead author, TS.

The ontological perspective for this study originally began as one of realism; however, with increased learning of the theoretical underpinnings of qualitative research, thematic analysis processes and the use of a reflective diary, this viewpoint became a more critical realist perspective; still acknowledging that reality always delimits what is possible (Sims-Schouten et al., 2007); but aware of the influence of the lens of interpretation held, and the contextual factors where the knowledge gained was situated in. Hence, realism would no longer be appropriate given the knowledge of, and reflection undertaken on, such mind-dependent truths (Tebes, 2005). For the TS, when carrying out the interviews and subsequent analysis, he needed to be aware of the need for reflexivity, given his commitment to the value and benefits of occupational therapy, and how such beliefs may impact the interaction with and analysis of the data. Moreover, the consideration of the viewpoints of contextually-situated professionals, the professionals’ “truths” may not be the same for adults with learning disabilities and informal carers. Additionally, given that this was undertaken in only one local authority, the organizational context must be considered as a potential influencer in responses and thus is not mind-independent.

Reflexive thematic analysis allowed the development of this theoretical perspective that grew with TS’s learning rather than a rigid one from the outset, which had to be adhered to, and the analytic process allowed a flexible back and forth between the different phases, which was important in order to promote a deeper understanding of the data. Moreover, as TS’s confidence grew with the analytical process, it allowed re-examination of the data to further investigate and reflect on the patterns that could be interpreted.

The analysis involved the following stages: familiarization; coding; generating initial themes; developing and reviewing themes; refining, defining and naming themes; and writing up. During the familiarization this involved listening to the audio of interviews and reading transcripts, including making notes (via the Memo function on NVIVO) and writing thoughts in a reflexive diary on this process. Coding the transcripts using NVIVO, TS identified meaningful segments/lines of interest and often listened to audio-recordings simultaneously to help bring the transcripts to life. In developing and reviewing themes, TS swapped to pen, paper and coloured pencils, which provided more insights before then using NVIVO again with colour schemes. Refining, defining and naming themes was greatly helped through writing “mini-abstracts”, which helped to identify core components of themes, whether they were in fact sufficiently different from one another, and as to whether such themes had rich enough content to constitute a theme. Writing up helped to develop this further; combining themes with quotes helped to ascertain the quality of the themes identified and whether further refinement—either in content or in name—was required.

### Ethics

This study was approved by the West Midlands—Coventry and Warwickshire Research Ethics Committee (REC Reference: 23/WM/0109), and then at a subsequent research governance panel at the participating local authority. All participants were required to provide written, informed consent before taking part.

## Findings

The following themes were conceptualized through analysis of the data of eight semi-structured interviews with occupational therapists from one local authority.

In brief, nine themes were identified; (1) Inclusive Practitioners; (2) Communication Difficulties Promote Uncertainty; (3) Informal Carers as Guides; (4) Hearing Informal Carers; (5) Occupational Therapists as Diplomats; (6) Moving and Handling Risk Managers; (7) Promoting Community Engagement; (8) Reducing Carer Burden; and (9) Workload Narrows the Scope of Practice.

(1) Inclusive practitioners

Occupational therapists made a variety of efforts to accommodate the needs of adults with learning disabilities in attempting to include them in the occupational therapy process.

In preparation for initial assessments, occupational therapists looked to organize them with the adult with a learning disability in mind, being aware of the time of day to visit a person to ensure their capabilities were such that they were going to have the best chance of being involved in discussions:

*“…if a person goes to a day centre during the day then we’d arrange it sometimes for when the person comes back home but maybe giving them a little bit of time to relax first, making sure they’re not too tired and they’ll be able to sort of participate in the assessment to the best of their ability” (OT008)*

Preparation for initial assessments also involved conversations with the informal carer in advance, to understand the level of communication an adult with a learning disability may be able to demonstrate; thus, helping occupational therapists to be aware of potential levels of participation in a conversation:

*“..can they talk and [the informal carer] say no, not really…they’ll make these noises, or that if they’re not happy or they can say this word and they can say that word and that kind of demonstrated that they’re happy or sad…” (OT004)*

During the assessment process, occupational therapists attempted— despite significant communication difficulties—to include an adult with a learning disability in discussions, directing their attention, conversation and body language towards a person:

*“she’s non-verbal, but I still talk to her and touch her and make sure she knows I’m there (OT003)”*

In follow-up visits, occupational therapists made concerted efforts to improve the chances that an adult with a learning disability may understand the type of support they may be providing, using more accessible forms of information and bringing in equipment to foster a more familiar relationship with it, and thus, a more sensory experience.

*“Using pictorial aids, sometimes if communication is a difficulty for the person. So for example, if we are talking about maybe using a hoist, to bring a picture of a hoist along with a person in it to show them how it’s used, so they get that understanding about what we are aiming to look at doing in regards with transfers and moving and handling” (OT008)*

*“actually having the equipment there…a visual…having the person touch it to know that it’s okay, to feel okay…” (OT003)*

Occupational therapists also made efforts to include the person’s views in the suitability of equipment for their needs—it wasn’t simply a professional dictating what equipment a person required—and a core part of this was seeking feedback through trialling it; occupational therapists were receptive to this and heeded the person’s wishes, for example, when a person may not like a piece of equipment:

*“…Is it the feel of..? Do you not like the sling? Do you not like the feeling of being hoisted?...Why is it not working for you? So it’s going through that process…I think it’s been months and months where you try different things” (OT005).*

(2) Communication difficulties promote uncertainty

Despite the concerted efforts (and ethos) of occupational therapists wishing to include an adult with a learning disability in the occupational therapy process, there was still unease about how they weren’t able to fully do this as a result of communication difficulties between them. There were particular feelings of uncertainty around: (1) truly knowing what the adult with a learning disability’s views were, including the impact of any intervention, (2) their skillset to deal with such communication difficulties.

Occupational therapists acknowledged that, in reality, much of the information they received to work on the needs of an adult with a learning disability came from the informal carer; as they could not access this information owing to the significant communication difficulties between themselves and the adult with a learning disability; and this was despite their awareness of the need to include them, preparatory work and consequent efforts to include the person as best as they could. Although this was a normal, routine part of practice, it was regularly noted about how this was uncomfortable for them, as they wondered whether the views of the carer, and indeed their professional viewpoints, matched with what the person’s needs truly were:

*“Unless you know, perhaps you have a whole kind of life history and you're very clear on what's of interest to the person. What are their wishes? And that is going to be more difficult with somebody who, where there's a difficulty with communication and also as we go through life anyway, things change, your wishes and what you like and maybe assumptions are made along the way”* (OT003)

Such uncertainty extended to the adult with a learning disability’s experience of any intervention. Occupational therapists felt that, in general, they had made a difference to their lives; but, uncertainty remained about what such interventions would *mean* for the adult with a learning disability or make them feel. Although responses on the importance of their work were given, the interviewer had to regularly probe such responses for further details, with sparse concrete examples given, and it appeared that some occupational therapists found communication difficulties as real barriers to identifying whether their intervention had really made a difference to the adult with a learning disability:

*“But some people aren’t able to express their gratitude or tell you how it’s improved their life, so I suppose you don’t really know from the person” (OT008)*

Occupational therapists identified in the interviews that they knew it was important for the adult with a learning disability to be heard; to understand their beliefs and values as part of occupational therapy work. But when communication difficulties arose—particularly those when communication difficulties appeared significant (e.g., where someone was non-verbal or there was no indication of a level of communication the occupational therapist understood), they did not know how to take this further; they had no resources to draw upon to formulate how they might approach communicating with this person. There was no indication that the occupational therapists felt satisfied that they had tried and concluded that the person was unlikely to give them information; they had tried, but without the full understanding of what *to* try, leaving them with uncertainty over their abilities to garner the person’s needs from the person themselves. A quote from OT007 puts this succinctly:

*“I just worry that am I getting to the root of it, have I asked the right questions, am I approaching it in the right way and teasing out…it could be that I’m missing something. Quite frankly if I’d have communicated in a different way and knew more than I do on the subject I’d be able to get to communicate with them better” (OT007)*

(3) Informal carers as guides

Given the communication challenges, occupational therapists had to find another way to support the identification of the needs of the adult with a learning disability; the discussions with informal carers, who knew the person best, provided the best “proxy” way to understand a person’s needs.

*“I think it is really difficult because we want to make the right decisions for somebody and, yeah, when you’re not getting a verbal response it’s very difficult to make a decision, so I think you have to lean quite heavily on the response from the informal carer and the decision is made with them.” (OT008)*

In addition to obtaining information from the carer themselves, informal carers were also able to act as a communication guide for the occupational therapist; someone who knew the person well and could help the occupational therapist interact with the person; this may have been through using language that would support the person to undertake an activity.

*“So there’s really no eye contact, there’s very little, I will be then saying to the carers who most of the time are the parents, ‘Could we try and get him to go into the bathroom? Could you show me how he sits on the toilet, etc, etc?” (OT002)*

or promoting understanding:

*“But sometimes the informal carer will say to the person ‘Isn’t that right?’ and then they might smile or laugh in response to that, so it’s like maybe they have got some understanding there. So it’s just gauging whether…how they’re responding to what the informal carer is saying and seeing how that feels really.” (OT008)*

(4) Hearing informal carers

Informal carers were not just sources of “proxy” information or communication supporters for occupational therapists in working with adults with learning disabilities. Their *own* views were taken seriously, and occupational therapists were attentive to these views. This was evident through the way in which occupational therapists described the time allocated to discuss matters with informal carers, both in terms of availability to discuss issues and in terms of the length of time allocated to have discussions with them. This enabled occupational therapists to feel that they had “heard” the informal carer, rather than just being passive recipients of information.

*“I allow quite some time if it’s appropriate, but just to sit and listen to that person, so it’s not a carer’s assessment per se, but I think if the person feels valued, the person feels respected, the person feels validated and acknowledged I think that that…it’s just a good foundation for a good therapeutic relationship that is reciprocal. So it’s putting just a little bit of legwork in at the beginning” (OT006)*

(5) Occupational therapists as diplomats

Although occupational therapists worked to make informal carers feel heard, they described that sometimes there were differences of opinion, and it required diplomatic efforts to support collaboration with them.

Occupational therapists had a mindset of collaboration in the face of disagreement, attempting to show understanding of the carer’s point of view:

*“because it’s so important to get the informal carer to try and trust you. Why should they? You are a representative of the local authority, some people find that quite a threat, so it’s delivering care in an honest, transparent, open way but with the carer feeling that they’re not threatened. I’m not there to contradict them, I’m not there to criticise them, I’m there to work with them.” (OT006)*

When faced with disagreements, occupational therapists applied awareness of the informal carer’s family context to help understand what may be beneath the reluctance to heed advice; parents who had looked after their (now adult) child for so many years:

*“You know, then they know their child better than anything, it can be challenging though because they again, they'll just kind have been doing this for many, many years, so kind of listening to advice and stuff can be hard for them. So in terms of trying to get through to them, sometimes is again it's a trial thing. So you know like, yeah, I’m listening to you about this, but let's just trial this first or okay, let's trial this and see if it works.” (OT004)*

When concerns arose from the informal carer, occupational therapists described the use of trialling equipment and adaptations as a diplomatic vehicle for supporting change, helping to reassure and persuade carers of their usefulness:

*“I suggested that we should go to the showroom and try, she was more than capable to use it. A few things that we have to think about that comes down to risk assessment. So the mother thought ‘Yeah, that is fine’, you know, ‘I can see she can use it’, so … just demonstrate.” (OT001)*

(6) Moving and handling risk managers

Occupational therapists highlighted their significant role in improving safety for adults with learning disabilities and informal carers via their risk management of moving and handling practice.

*“I’m hoping that with the information we give them about how to do things, how to move somebody in a slightly safer manner, how to get somebody up from the floor maybe and using equipment correctly makes them safer and less likely to have an injury… (OT002)*

When occupational therapists perceived that risky moving and handling practice was being undertaken, it appeared that it was always the informal carer with whom this issue was discussed with, and this was undertaken in a supportive way in the first instance:

*“…, I'm sure I could see why you [the carer] would think that, however, then maybe talk about the risks, because they might not have thought about the risks in that, and shall I just do a risk assessment, a management plan, it’s like they are continuing to do something which is maybe against advice. I think as professionals as well, we need to understand the reason why they're doing that. I mean, nine times out of 10, I've never seen anybody do it purely because they're trying to be neglectful or abusive. It’s normally because of, you know, they've never been shown in a different way. Or they're just used to doing it that way, you know. So if you kind of highlight the risk and stuff, but again, in a nonjudgmental way …” (OT004)*

However, this was the area where collaboration between occupational therapists and informal carers could break down, requiring external personnel and safeguarding procedures to be initiated. Underpinning this was the risk to the adult with the learning disability (who often did not have the capacity to consent to whatever moving and handling practice was being advised against), and thus a greater level of authority was summoned to support safe practice:

*“I mean if somebody doesn't want to accept equipment and doesn't want to accept your recommendations, informal carers, then we have to have, sometimes have those difficult conversations about actually, this is what could happen and kind of go into that and whether it potentially it's going to end up, you know, under safeguarding investigation, that's the other thing you know.” (OT003)*

*“I would explain, I’d communicate with the person, I’d go and visit and show them where the risks are and help them to understand how the risk might be affecting them and the person involved and that we’re trying to work in the best interests of the person and to help their understanding, to help them to make…to understand our point of view, but if they’re still not understanding or still refusing then you have to escalate.” (OT008)*

(7) Promoting community engagement

Beyond their role as risk managers, occupational therapists described how interventions helped to promote individuals’ abilities to access meaningful activity beyond the home, including day centres and the community more broadly.

The occupational therapist outlined adaptations that directly promoted access to/from the home, e.g., via ramping to make the entrance wheelchair accessible. Such an intervention meant that not only would the community be more physically accessible to them, but it could have a beneficial impact psychologically with participation in subsequent community activities that were now more accessible to them.

*“Even if somebody can facilitate access into…they can go back to a day centre, they can go for a walk around the park, they can go and have an ice cream in the park, that they can enjoy being outdoors. We know the impact of being outdoors has on somebody’s health and well-being, it just opens the floodgates doesn’t it of being able to, ‘I can’, so if they can’t then the negative spiral.” (OT006)*

Occupational therapists provided equipment to support moving and handling both within the home and within the community setting, meaning that adults with learning disabilities would be more likely to be able to be supported to participate in such settings.

*“I think enabling people to not just be sat in chairs and beds, enabling people to access the community you know, to go to day service, to access activities, just through supporting with recommendations for how the transfer is going to be done. What a day service, managing, you know how they're going to manage their personal care needs there because it's complicated, supporting staff there to be confident about it and perhaps have recommendations for good and bad days?” (OT003)*

Finally, occupational therapy intervention had a more indirect impact on supporting engagement beyond the home environment; through improved accessibility (e.g., via adaptations) with full-washing and improving levels of hygiene. Occupational therapists described how improving a person’s level of hygiene meant they would be more likely to access the community through an improved sense of self-esteem:

“*Also I had a client who had a learning disability who had a shower over the bath which wasn’t at the right height and we got it replaced so that he could have a shower. Because his behaviour was so impacted by not being able to shower properly and his hair wasn’t washed for weeks and months and then he couldn’t go to college because he felt embarrassed that he wasn’t clean and so by putting this adaptation in place his behaviour…he was just becoming really annoyed and frustrated so his behaviour was getting worse. So then this putting the shower in over the bath resolved the issues and his behaviour is much better, he can carry on with his routine and access college again” (OT008)*

(8) Reducing carer burden

Occupational therapists described how their interventions benefited informal carers by reducing carer burden, improving their ability to carry out their caring role, which may have been more difficult in the past:

*“Yeah and quite often they will say ‘Oh this has really helped me, thank you so much, it’s so much easier now to manage with this adaptation in place, or this equipment or in this new property, yeah.” (OT008)*

A number of occupational therapists noted that some of these benefits were due to the adult with a learning disability being able to do more for themselves, particularly after the provision of a profiling bed. Thus, in some situations, the improved independence of adults with disabilities meant that less informal care could be required, with carers then having additional time for their own occupations:

“*And he was able to use the handset and things independently as well, so it wasn’t as if she needed help with, okay, let’s pull you up the bed now because you’re moving further down, he could use the handset on his own so I think it gave her a lot more freedom, especially in the mornings*” *(OT005)*

More broadly, occupational therapists discussed how their input improved informal carers’ psychological health and ability to cope:

*“… If you’re making things easier in a physical sort of sense and it’s less stress for the person and it obviously affects their well-being if they’re distressed all the time and not coping, they could have a breakdown if they’re not able to cope, so any support we can put in place can help benefit the person and help their mental health as well.” (OT008)*

(9) Workload narrows the scope of practice

Occupational therapists described a want to improve the breadth of their involvement with adults with learning disabilities and their informal carers, but this was limited by the workload pressures upon them. The volume of work meant that occupational therapists would direct their attention to the current problem a person was having, being “in and out” before moving on to the next case, without exploration of other wider occupational areas that could improve a person’s quality of life. Occupational therapists were pragmatic about this situation—feeling they simply could not explore these areas with the limitations on their time, but their views illustrated a desire for this situation to change—that their role (and its consequent impact) could do more to improve people’s lives:

*“I suppose looking at maybe the bigger picture as well, I think you tend to focus on what the problem is at that, you know at that stage because again, people normally come in at that point, but it would be so much nicer and better to be … actually look at the bigger things, and that stuff that they can do which could really, really kind of change their life. If you're just kind of concentrating on the essential stuff, that seems to happen at the moment.” (OT004)*

There was little consensus about what the “wider” role could be, but there was agreement that the remit of their current role was too narrow. Interestingly, occupational therapists didn’t really identify that these areas were identified and not acted upon; rather, they highlighted that conversations about such areas may not be happening in the first place, and their workload was the culprit:

*“…are we apprehensive about asking too many questions because when we do we could be expected to work in ways that we just can’t. I think we’re under so much pressure. If you start asking questions do they want more and more? I don’t know really” (OT008)*

## Discussion

This study was a small, explorative piece of research that has helped to elucidate the experience of social care occupational therapists working with adults with learning disabilities and their informal carers. To the authors’ knowledge, there has been no previous published research on this topic; thus, this study contributes evidence to an area previously not documented. Importantly, the design, including the research questions, was co-produced with experts by experience and social care professionals, enabling the focus to be on multiple areas regarding what really mattered to those who receive and deliver social care services: communication, collaboration and making a difference as part of social care occupational therapy practice.

Occupational therapists made concerted efforts to facilitate communication with adults with learning disabilities, working inclusively—taking into account their needs—in order to provide them with an environment and/or resources that supported their communication (and potential collaboration) as part of the occupational therapy process. This included ensuring that visits were held at an appropriate time of day for the person, asking the carer about what a person’s responses might mean, directing their attention to the person, using visual and tactile information—particularly with the use of equipment—and seeking feedback about such equipment. Such behaviour would be concordant with the various legislative areas that aim to support such inclusive practices, including the Care Act 2014 and c. 23 (2014), the Equality Act (2010) and the Mental Capacity Act 2005 and c. 9 (2005).

It is notable, however, that despite the above actions, occupational therapists’ psychological state in such interactions is filled with uncertainty; they feel that when significant communication difficulties are apparent, their professional viewpoint and the perspective from informal carers (which influence such professional views) are assumptions, and may not be in fact, an accurate reflection of what an adult with a learning disability truly wants. Underpinning this was the identification of their limited skillset in navigating such communication difficulties. This is echoed in the literature, often in studies with support workers, where a lack of knowledge about ways to support communication with adults with learning disabilities contributes to the difficulties in interactions (see, for example, Dalton & Sweeney, 2013).

There will be situations where an adult with a learning disability and significant communication difficulties will not have the mental capacity to make a particular decision about their care. This is where the informal carer would likely be the justified primary source of non-professional influence on a person’s assessment and intervention, which is much needed and historically has not been the case (Care Quality Commission, 2016). Yet, the opposite can also be true, and an adult with a learning disability, despite such communication difficulties, will in fact have the mental capacity to make a particular decision. In ascertaining this, it is evident that occupational therapists need more support. As noted by Sevcik et al. (2022), professionals must *not* judge the complexity of the thinking skills of adults with learning disabilities solely based on their expressive communication skills; their receptive skills are often more developed, and thus, they may *understand* more than they can communicate. Accordingly, this is where additional training could help upskill occupational therapists in potential ways to support them. In addition, the development of collaborative pathways with speech and language therapists—including where there are opportunities for the employment of these therapists within local authorities themselves—would likely be the most fruitful of support options given their expertise, which includes supporting mental capacity assessments in people with communication problems (Jayes et al., 2021).

Occupational therapists relied heavily on the guidance of informal carers. This included helping the occupational therapist to communicate with the person and understand responses, but primarily it was the direct information provided by carers to inform occupational therapy assessment and intervention. Previous work by Glendinning et al. (2015) noted the use of informal carers as a ‘resource’ but, despite improved rights, not as a co-client. Contrary to this, the present study has highlighted that, as well as being a source of information, occupational therapists placed great importance on making carers feel heard, with an empathetic listening ear being utilized to value informal carer views as part of the occupational therapy process.

Collaborative efforts extended to where disagreements with informal carers were occurring, where occupational therapists acted as compassionate negotiators in such situations; diplomatic efforts to work *with* the carer rather than utilizing an authoritarian approach. Moreover, there was an awareness of the impact of long-standing parental relationships, with parents in a ‘protective’ role being an important consideration as part of this. Occupational therapists navigated such situations through the use of trialling equipment; having informal carers observe what they thought might not work, or be too risky, helped to open their eyes to new, acceptable ideas that could benefit the adults with a learning disability they cared for. Such practices can be seen as being risk-positive or risk-embracing, exploring possibilities with creativity and also resilience (Seale et al., 2013), and not being afraid to try new things in pursuing an improved quality of life for an adult with a learning disability. Overall, the above highlights the importance of social care occupational therapists having numerous skillsets in empathetic listening, as well as creative and resilient positive risk-taking, and which is underpinned by a diplomatic manner that aims to navigate the concerns of informal carers to move into uncharted occupational territory.

Occupational therapists emphasized this collaborative rather than dictatorial approach in managing moving and handling risks, aiming to improve transfer safety for both adults with learning disabilities and their informal carers. The Health and Safety Executive (2026) underscores that moving and handling risk assessment protects both care recipients and providers, as poor practice can lead to injury, accidents, and loss of dignity. As such, in line with the Care Act 2014 and c. 23 (2014), such assessments are central to wellbeing. Although research on moving and handling by informal carers for adults with learning disabilities is limited, existing literature highlights that such informal caregiving is linked with increased musculoskeletal injuries (Darragh et al., 2015), with Wyton (2018) noting specifically about the negative health outcomes where moving and handling risks have not been addressed. Consequently, social care occupational therapists must demonstrate confidence and competence in moving and handling risk assessment to protect both parties.

Collaboration between occupational therapists and informal carers can break down when carers resist recommendations to address moving and handling risks, particularly where the adult with a learning disability lacks the capacity to consent to such risks. In these cases, informal carers are making moving and handling decisions that may contravene what the occupational therapist perceives to be in the individual’s best interests, and where the adult with a learning disability may need to be safeguarded owing to being at risk of harm (e.g., neglect) as a result of not receiving recommendations. This underscores the need for occupational therapists to be legally literate in applying relevant social care legislation, such as the Mental Capacity Act (2005) and Care Act (2014), whilst demonstrating assertiveness in challenging unsafe practices.

Beyond managing risk, occupational therapists identified other ways in which their role helped make a difference in people’s lives. For adults with learning disabilities, this centred around promoting community engagement. This was demonstrated through interventions addressing barriers to such engagement, e.g., adapting the home environment to promote physical access (e.g., via ramping for wheelchair access) or moving and handling solutions to allow someone to be supported (and therefore access) day centre provisions. Moreover, a psychological impact was noted in relation to hygiene; adaptations that promoted a full wash meant improved cleanliness and thus improved self-esteem in social situations. Such community access may have multiple, far-reaching implications; being able to (and *feeling able to*) access your local facilities and resources (thereby meeting one of the needs identified by the Care Act, 2014) could include participation in all manner of meaningful occupations, promoting their occupational rights (Reep & Ramsey, 2023). Moreover, it would also support attendance at various health appointments in a community setting—particularly important for adults with learning disabilities who have poorer health than the general population (Wigham et al., 2022).

However, it would be remiss to note that the occupational therapists in this study identified the uncertainty of knowing about whether they would make a difference in people’s lives and, as such, these outcomes—particularly without collateral information from adults with learning disabilities and/or informal carers, are assumptions—and the full extent of any impact of such intervention is speculative based on the judgements of a professional viewpoint. The potential for improved communication through additional training and inter-disciplinary working (as noted above) could mean that occupational therapists become more aware of, or confident in, the impact their work may be having. In addition, there may be psychometrically tested measures that do not rely on complex verbal communication (e.g., the Model of Human Occupational Screening Tool [Parkinson et al., 2006]), which could improve the gathering of the impact of occupational therapy work, which would not only help highlight the potential benefits of occupational therapy, but also provide more of a ‘voice’ to those adults with learning disabilities. Finally, a mindset-change may also be required, particularly when working with those adults with severe and profound learning disabilities; focusing not on the benefits of occupational therapy as empowering independence, but rather, an acknowledgement of self-determination for those individuals who are inherently dependent (Skarsaune et al., 2021).

There is seemingly more clarity regarding the effect on the lives of informal carers, who are more able to report to occupational therapists about the impact of any intervention on their lives, including reducing carer burden. Although caring is an idiosyncratic experience, and there are of course those who feel that ‘burden’ may not fit their viewpoint of what their caring role is, legislation (e.g., the Care Act 2014 & c. 23, 2014) and research (Rand & Malley, 2014) indicate the potential detrimental impact of caregiving on health and well-being and thus the need for carers to be supported. Professionals felt that the introduction of new equipment and adaptations meant that the burden on caring is somewhat reduced, meaning that carers can provide care with greater ease than before. Occupational therapists also highlighted various areas of intervention that could also mean more time for the informal carers to pursue *their* occupation. The use of profiling beds—which provide mechanical means to improve independence and support safer moving and handling practices—appears to be a key aspect of provision in improving the independence of those receiving care, and simultaneously, potential additional time for informal carers. Occupational therapy intervention can also improve access to day services, which could have significant knock-on effects for informal carers in addressing their occupational needs, with a consequent impact on their health and wellbeing. Day centre use would reduce the number of informal carers’ care hours—which is linked to improved satisfaction with life and health-related quality of life (Pennington et al., 2025). Underpinning this may be the increased opportunity to engage in other meaningful occupations, for example, social activities, or employment; reduced engagement in social activities is known to contribute to carer burden (Liu et al., 2020), while employment is frequently disrupted or limited by caregiving responsibilities (Carers UK, 2023).

Finally, occupational therapists reported having a significant workload alongside established expectations of the remit of the occupational therapist role. This limited their ability to explore wider occupational areas within their practice. It was not evident from the interviews that occupational therapists had a shared vision of what their wider role might be, and pragmatically they felt unable to go beyond their current remit, but their responses indicated a desire, theoretically, to expand the social care occupational therapy role they were undertaking to take on a broader scope of occupations when working with an adult with a learning disability and their informal carer. Thus, it appears as if there is “untapped potential”, which, with the right resources, it could mean that adults with learning disabilities and informal carers could benefit further from their expertise across a wider range of occupational areas. Although this perhaps is unlikely given the economic climate of local authorities being asked continually to do more with less (Gray & Barford, 2018) and has been historically recorded as an issue in occupational therapy since the 1980s (Scrivens, 1983); the Care Act 2014 and c. 23 (2014), with its focus on a variety of outcomes—meaningful to the person, could mean that widening the scope may, eventually, be a possibility with improved resources, or technological innovation (e.g., the use of artificial intelligence) which frees up practitioners to carry out their work.

## Limitations and further work

Professional viewpoints are key in understanding the delivery and impact of services and a significant element of a co-productive approach. However, so are the views of those people actually in receipt of services; adults with learning disabilities and informal carers. This was not possible as part of this research study, and accordingly, the themes elucidated after analysis are professional perspectives and should not be taken as the full picture in this area of work. Further work should attempt to gather the views of these populations in order to promote a fuller, more rounded understanding of communication, collaboration and make a difference as part of social care occupational therapy delivery.

In addition, it is worth noting that as learning disabilities can vary significantly, for those adults with learning disabilities with less significant communication problems, the experience of occupational therapy provision for both professionals and adults with learning disabilities and informal carers may be very different, given the strength of the impact of communication difficulties throughout the themes identified. Moreover, certain elements of the findings, such as those relating to moving and handling practices and risk, are much more likely to be applicable to those adults with significant physical problems. Other studies should therefore investigate practices for those with fewer communication barriers or less complex physical needs.

Finally, given the small sample size within a single local authority, additional qualitative or mixed methods research is needed across a wider sample of local authorities. This would help to identify the commonalities of the patterns of meaning described and whether considerable variation exists. Such work is important to identify the scale of implications; e.g., if such themes are prevalent across local authorities in England, it may indicate a more national approach to initiatives to build on strengths and improve areas of development.

## Conclusion

Social care occupational therapists aim to be inclusive in their practice for adults with learning disabilities, and informal carers are seen as both guides and individuals who need to be heard in their own right. However, an uncertainty lurks beneath the surface, with significant communication problems creating a reliance on informal carer input and occupational therapists having doubts over their abilities to navigate such communication problems. This leaves lingering worries about not knowing what the wishes of adults with learning disabilities truly are, and the level of impact an intervention has on them. Communicative and collaborative efforts may be able to be improved through further training or liaison with other specialist professionals (e.g., speech and language therapists). Occupational therapists appear to be able to successfully traverse disagreements and remain collaborative with informal carers to support positive-risk taking with adults with learning disabilities, but issues arise when moving and handling practice presents risks and recommendations are not heeded. This highlights the importance of legal literacy and the ability to utilize diplomatic and assertive skillsets to best support both adults with learning disabilities and informal carers. Beyond risk management, social care occupational therapists appear to have an impact on promoting community engagement for adults with learning disabilities and reducing carer burden for informal carers, highlighting a professional role that is integral to the purposes of the Care Act. Yet, there is a belief that their scope of practice is limited, and with more resources, their impact could be even greater.

## Data Availability

The participants of this study did not give written consent for their data to be shared publicly, so, owing to the sensitive nature of the research, supporting data is not available.
